# BRD4 represses developmental and neuronal genes through interaction with PRC1.6

**DOI:** 10.64898/2026.01.31.702994

**Published:** 2026-08-05

**Authors:** Fanny Boulet, Manthan Patel, Zeinab S. Zanjani, Nuria Andres-Sanchez, Anam Ijaz, Debosree Pal, Pankaj Dubey, Aoife Murray, Dean Nizetic, Michael D. LeClaire, Karina L. Bursch, Brian C. Smith, Madapura M Pradeepa

## Abstract

BRD4 is best known as a transcriptional co-activator, yet heterozygous loss-of-function variants cause craniofacial and neurodevelopmental abnormalities through unclear mechanisms. Using human embryonic stem cells and neural organoids as an in vitro model of human embryonic brain development, we show that BRD4 represses polycomb-regulated developmental and neuronal genes. BRD4 co-occupies bivalent promoters with PRC1.6 and directly interacts with PCGF6 and RING1B through its C-terminal domain. H3K23ac contributes to BRD4 recruitment through BD2, linking acetylated chromatin recognition to polycomb-associated repression. Acute BRD4 depletion alters PRC1.6 occupancy, reduces ubiquitination of H2AK119 and rapidly increases developmental gene transcription. In neural organoids, BRD4 BD2 mutations alter neuronal cell composition and increase expression and chromatin accessibility associated with neuronal transcription-factor programmes. These findings establish BRD4 as a context-dependent chromatin regulator that prevents premature activation of developmental genes and provide mechanistic insight into BRD4-associated neurodevelopmental disorders.

## Full Text Availability

The license terms selected by the author(s) for this preprint version do not permit archiving in PMC. The full text is available from the preprint server.

